# Gelatinized Cassava Starch Obtained via Low Molar Ratio Hydroxypropylation Reaction

**DOI:** 10.1021/acsomega.5c00246

**Published:** 2025-03-20

**Authors:** Henrique Solowej Medeiros Lopes, Fernanda Andrade Tigre da Costa, Daniel Komatsu, Alain Dufresne, Aparecido Junior de Menezes

**Affiliations:** 1 Federal University of São Carlos (UFSCar), 110 km João Leme dos Santos Road, Sorocaba, SP 18052-780, Brazil; 2 Technological College of Sorocaba (Fatec), 2015 Carlos Reinaldo Mendes Avenue, Sorocaba, SP 18013-280, Brazil; 3 Nuclear and Energy Research Institute (IPEN-CNEN/SP), 2242 Prof. Lineu Prestes Avenue, São Paulo, SP 05508-000, Brazil; 4 Pontifical Catholic University (PUC), 290 Joubert Wey St., Sorocaba, SP 18030-070, Brazil; 5 Université Grenoble Alpes, CNRS, Grenoble INP, LGP2, Grenoble F-38000, France

## Abstract

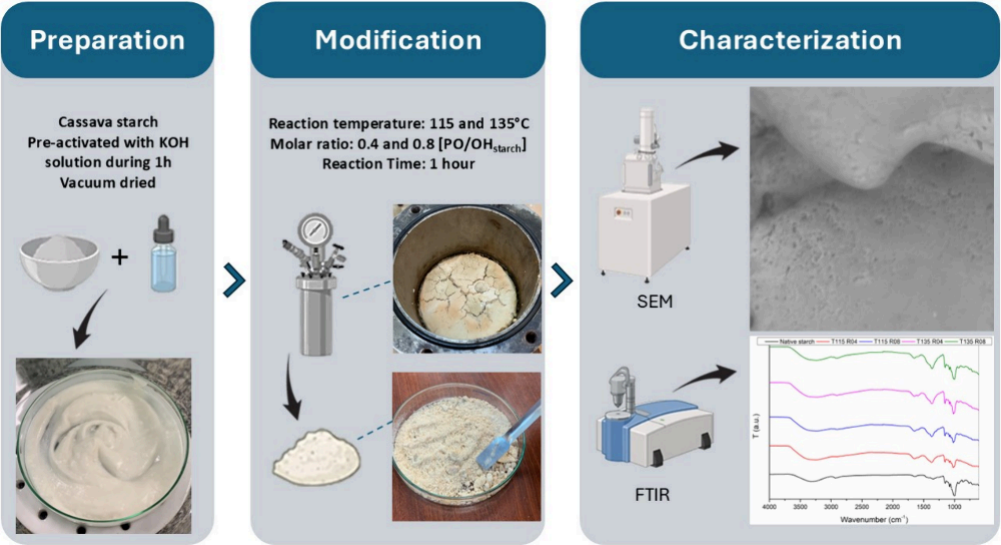

Hydroxypropylated starch was successfully synthesized, aiming to address the limitations of native starch, such as poor mechanical properties and water sensitivity, which hinder its application in biodegradable polymers. The modification process, conducted at 115–135 °C with propylene oxide (PO) molar ratios of 0.4–0.8 [PO molecule per OH of starch], effectively disrupted the native starch structure. FTIR and ^13^C NMR confirmed methyl group incorporation, with lower temperatures and higher PO ratios yielding greater modification. SEM and XRD analyses demonstrated complete gelatinization; although some short-range order structures are present, long-range structures were eliminated, while DSC confirmed the absence of gelatinization peaks. TGA revealed the integration of lower molecular weight molecules, suggesting PPO homopolymerization within the starch granules. These structural transformations enhance the feasibility of producing hydroxypropylated starch films with reduced plasticizer content and energy requirements, offering a novel approach to improving starch-based materials for biodegradable applications.

## 1 Introduction

Starch-based bioplastics are one of the most promising alternatives to conventional polymers in various market segments, such as short-term packaging and mulch films.^1^ Nevertheless, the intrinsic properties of starch limit its application. Its high sensitivity to moisture, retrogradation, and poor mechanical properties, combined with low processability, often make it difficult to use as packaging.^2^ This is why starch is commonly prepared by different approaches, such as blending,^3^ composites,^4^ or chemical reactions.^5^ Blending of starch-based materials usually occurs by melting processes, such as extrusion, with another biodegradable polymer that changes its mechanical properties and water absorption behavior, increasing elongation at break and improving barrier properties.^3^ Similarly, the production of composites is more focused on improved tensile strength and elastic modulus, by adding natural fibers, combined with a decrease in the water absorption behavior.^6^ Modification of starch can occur by several different methods, such as cross-linking, esterification, etherification, acid hydrolysis. Cross-linking reactions may produce a starch with lower swelling power, hindering gelatinization, which is the opposite observed for esterification or etherification processes, and acid hydrolysis produces a low-viscosity starch material with higher gel strength. Each one of them represents a different approach for a different application, from food to packaging.^1,7^

Structurally, starch is composed of two major macromolecules, amylose and amylopectin. Amylose is a predominantly linear polysaccharide, composed of α(1→4) d-glucose units with a molecular weight around 1 × 10^6^ g mol^–1^. Amylopectin is a branched polysaccharide, also composed of α(1→4) d-glucose units with branching points at α(1→6) and molecular weights around 50–500 × 10^6^ g mol^–1^. These macromolecules are radially organized, intercalating amorphous and crystalline regions, forming the starch granule.^8,9^ Starch granules are composed of three main crystalline structures, called A, B, and C, which depend on the starch source. The crystalline portion is related to branches of amylopectin and consists of double-helix chains in a hexagonal unit cell, varying according to the presence of water molecules within the structure. To obtain a thermoplastic material, the semicrystalline structure of starch must be disrupted by a common process called gelatinization (also called plasticization), under the effect of heat and in the presence of a plasticizer.^8−11^

**Scheme 1:**
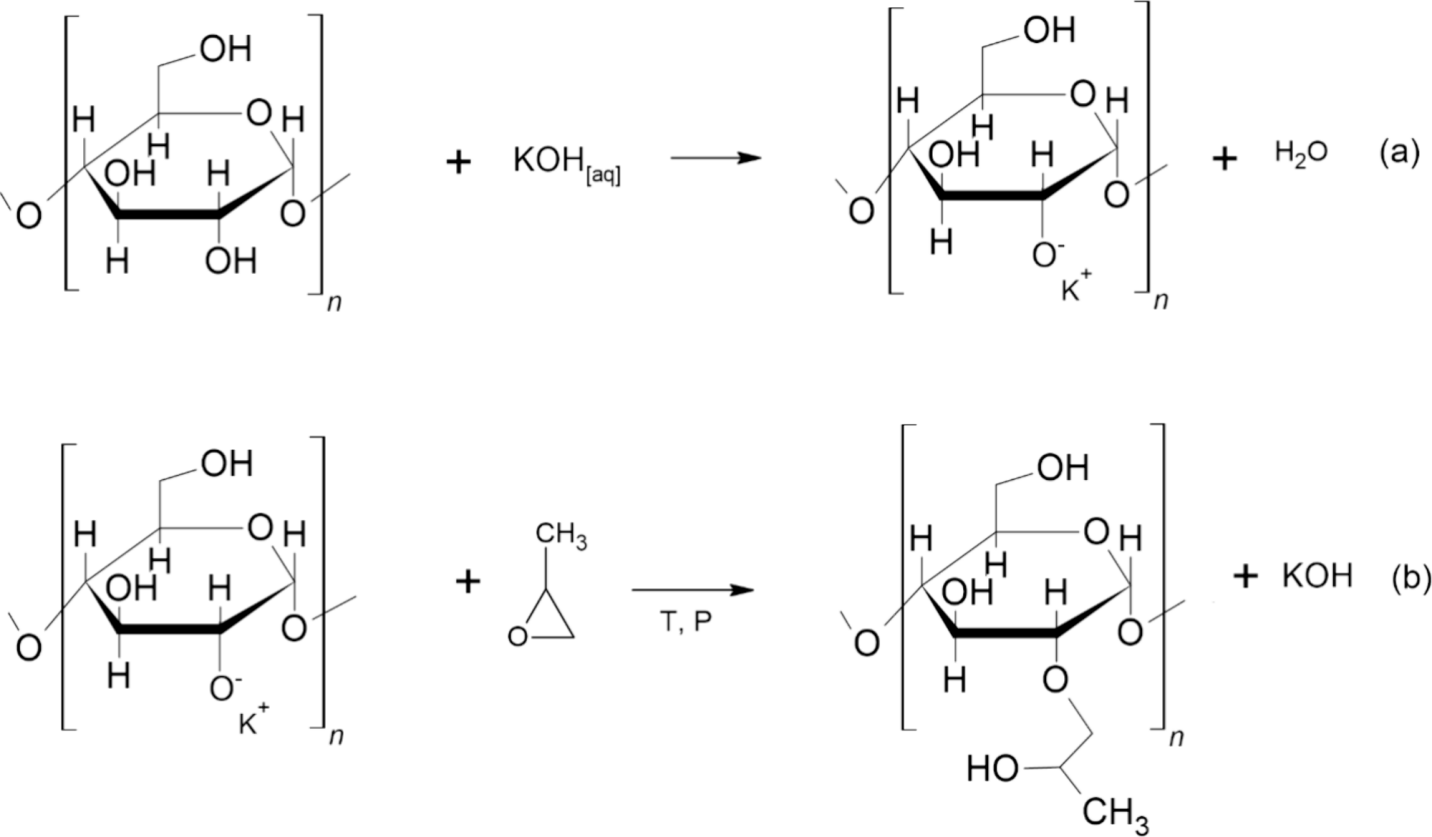
Activation of the d-Glucose Unit with Aqueous KOH (a) and Modified under Temperature, Pressure, and the Presence of PO, Forming Hydroxypropylated Starch (b)

Since conventional methods for preparing thermoplastic starch (TPS) exhibit issues, alternative methods have emerged as a possible solution. One example is hydroxypropylation, which involves the complete or partial substitution of hydroxyl groups with propylene oxide (PO) molecules. The benefits of this process for preparing starch-cellulose composites are well documented in the literature.^5,12−14^ In some studies, it has also been observed that hydroxypropylation can delay or prevent starch retrogradation due to steric hindrance caused by PO side groups. This prevents chain reassociation and increases the flexibility of starch films,^15−17^ combined with higher hydrophobicity.^5^ Also, it is known that during hydroxypropylation, some byproducts are formed, such as poly(propylene oxide) (PPO), a homopolymer formed from PO by chain transfer reactions, alongside the grafting of PO groups in starch chains, as presented in Scheme 1. As a side reaction, PPO can be formed, primarily due to the excess of unreacted PO molecules in the bulk under suitable conditions. This polymerization occurs via a chain growth mechanism, where a hydroxyl group opens the epoxide ring of PO, creating a new hydroxyl species that can react with additional PO molecules nearby, thus propagating the chain growth. This homopolymer can be identified as a lower molecular weight compound within starch chains with a very low *T*_g_ around −75 °C.^18,19^

Hence, in this work, hydroxypropylated starch (HPS) samples were prepared under high temperature and pressure using different molar ratios of 0.4 and 0.8 [PO/OH_starch_] and reaction temperatures of 115 and 135 °C, analyzing mass gain as an output of the reaction, alongside starch structure, extent of modification, and thermal properties. The novelty of this work is the obtention of a completely gelatinized starch after a low molar ratio hydroxypropylation by the homopolymerization of PPO and under certain pressure and temperature conditions, a feature not observed under similar hydroxypropylation conditions^5,12−14^ nor under different conditions,^19−25^ only being partially obtained under a high level of modification and/or shear rate conditions,^16,17,26,27^ as emphasized in a review of HPSs.^28^ The obtention of a full gelatinized starch straightforwardly after the reaction indicates that hydroxypropyl groups reached also the crystalline regions due to factors such as temperature and pH^29^ and could facilitate the route for obtention of starch-based materials, dispensing the need for an additional process and reduced or exempted use of plasticizers for product obtention.

## 2 Materials and Methods

### 2.1 Hydroxypropylation Reaction

Reactions were carried out in a stainless-steel autoclave equipped with a manometer. Temperature was monitored by a digitally controlled thermocouple heated by electrical resistance. Prior to the reaction, food-grade cassava starch (Pinduca, Brazil) was characterized regarding molecular weight^30^ and amylose content^31^ (shown in the SI), and values were about 1.52 × 10^6^ g mol^–1^ and 20.7%, respectively. Furthermore, starch was pretreated for 1 h with potassium hydroxide (Neon, Brazil), dissolved in a solution of 99.5% ethanol (Neon, Brazil):distilled water, in a 5:1 volume ratio. The mixture was then placed and sealed in the reactor with the addition of the desired amount of PO (Sigma-Aldrich, USA), according to the defined molar ratios (0.4 and 0.8). The mixture was then heated to different temperatures of 115 and 135 °C (Table 1), and the pressure rising was monitored using a manometer. All chemical reagents used were of analytical grade. A design of experiments (DoE) was conducted using Minitab statistical software, with two levels of each modification parameter to evaluate the statistical significance of the model using an analysis of variance (ANOVA). Reaction parameters were defined according to the literature^5,14,32,33^ and after several optimization trials.

**Table 1:** Experimental Design and Sample Design for Hydroxypropylated Starch

sample designation	molar ratio [PO/OH_starch_]	temperature (°C)
T115 R04	0.4	115
T135 R04	0.4	135
T115 R08	0.8	115
T135 R08	0.8	135

The reaction time was defined as 1 h, and a typical pressure per time curve is available (shown in the SI). The system was then cooled, and the product was removed. Samples were investigated in triplicate for bulk mass gain, and pH was measured from 1% suspensions, right after the reaction. Soxhlet extraction was further performed during 12 h with *n*-hexane for all samples.

### 2.2 Characterizations

Fourier-transform infrared spectroscopy (FTIR) was performed on PerkinElmer Spectrum 100 (USA) equipment, from 4000 to 400 cm^–1^ and with a resolution of 4 cm^–1^, coupled to an attenuated total reflection (ATR) apparatus using 16 scans. For quantitative analysis of short-range structures, FTIR spectra were processed in absorbance mode. The data were adjusted and normalized to zero absorbance (0 A), followed by baseline correction using a straight line in the spectral region of 1200 to 800 cm^–1^. Subsequently, the peaks within this region were deconvoluted and optimized using Bessel function and the amount of short-range ordering in starch was calculated by the height ratio (measured from the baseline) at 1047, 1035, and 1014 cm^–1^, based on the essentially described by ref (34). Solid-state carbon nuclear magnetic resonance (^13^C NMR) was performed on an Avance III 400 MHz spectrometer with a Cross-Polarization/Magic Angle Spinning (CP/MAS) kit at 298 K and 12 kHz rotational speed. The relative degree of substitution (DS) was estimated by the integration ratio between the methyl and C1 signals at approximately 20 and 100 ppm.^35^ CHNS and elemental analysis (EA) were measured using a Thermo Finnigan Flash EA 1112 under 970 °C for both furnaces with a helium atmosphere. Samples were analyzed at least twice. DS was calculated based on the literature, according to eq 1.^36^
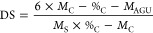
1where *M*_C_ is the molecular weight of carbon in the anhydroglucose unit, %_C_ is the relative carbon content obtained by EA, *M*_AGU_ is the molecular weight of the anhydroglucose unit, and *M*_S_ is the molecular weight of the substituted molecule. Morphological analyses were performed by scanning electron microscopy (SEM) observations, carried out on dry samples with Hitachi TM3000 equipment with an intensity of 15 kVA without metallization. Structural changes were investigated with X-ray diffraction (XRD) by a PANalytical X’Pert PRO MPD from 5 to 50° and a wavelength of 1.5418 Å. Quantitative analysis of long-range structures was determined by the crystallinity index (*X*_C_), according to ref (8) and expressed in percentage, using eq 2.
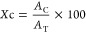
2where *A*_C_ is the area of crystalline peaks and *A*_T_ is the sum of the amorphous halo area with the crystalline peak area, deconvoluted from the curve. Differential scanning calorimetry (DSC) experiments were performed with TA Instruments DSC 25 (USA) equipment coupled with a refrigerated cooling system (RCS) with a nitrogen flow rate of 50 mL min^–1^. Measurements were performed from −90 to 120 °C at a heating rate of 10 °C min^–1^, and properties were identified by the software TRIOS. Thermogravimetric analysis (TGA) was performed from room temperature to 700 °C under a heating rate of 10 °C min^–1^ and an inert atmosphere in a TA Instruments (USA) SDT600 system with up to 10 mg samples.

## 3 Results and Discussion

### 3.1 Design of Experiments (DoE) and Analysis of Variance (ANOVA)

Regarding the reaction environment, pH values were not influenced by the defined parameters, such as temperature and molar ratio, and presented at 11.7 ± 0.3 for all samples.

Observed mass gain is higher for samples modified at a lower temperature (115 °C) with a molar ratio of 0.8. Table 2 shows the statistical dispersion (*F*-value) and significance tests (*P*-value) applied to the obtained mass gain values. It is possible to observe that only the molar ratio presented a *P*-value lower than 0.05, showing a high influence on the obtained mass gain. The opposite trend was observed for temperature, with a *P*-value higher than 0.05, reducing the effectiveness of the reaction, different to what was reported by ref (32) where the authors observed a similarity when increasing temperatures. On the contrary, an increasing molar ratio increases the mass gain, as expected and mentioned by others.^32^*F*-values accompanied the same tendency, with higher values observed for molar ratio differences. Preliminary tests showed that higher molar ratios (above 1) combined to higher temperatures (150 °C) converted the whole bulk in polyol, obtaining no solid material, different to those observed by refs (5,12−14) explaining the defined values at Table 1 and evidencing the capability of the reaction performed to modify the starch. In addition to mass gain analysis, tests showed that HPS does not present solubility in dimethyl sulfoxide (DMSO) and tetrahydrofuran (THF), even when tested under a high temperature (70 °C).

**Table 2:** *F* and *P* Values of Each Parameter Analyzed

source	*F*-value	*P*-value	mass gain (%)	
temperature	1.79	0.211	T115 R04	229.90 ± 13.63
			T115 R08	280.24 ± 19.66
molar ratio	9.16	0.013	T135 R04	162.06 ± 20.09
			T135 R08	254.00 ± 12.89

PCA results (shown in the SI) highlight the distinct effects of molar ratio and temperature on mass gain. Molar ratio positively influences mass gain, while temperature has an inverse effect, aligning with ANOVA observations. The first component, primarily associated with mass gain (0.707) and molar ratio (0.616), confirms the molar ratio as the key driver. Temperature plays a minor role (−0.347) but dominates the second component (0.871), indicating a separate influence. The third component captures a secondary trend where mass gain (0.707) and molar ratio (−0.616) sometimes vary oppositely. Overall, the molar ratio drives mass gain, while temperature affects the system differently.

### 3.2 Fourier-Transform Infrared Spectroscopy (FTIR) and Solid-State Carbon Nuclear Magnetic Resonance (^13^C NMR)

**Figure 1:**
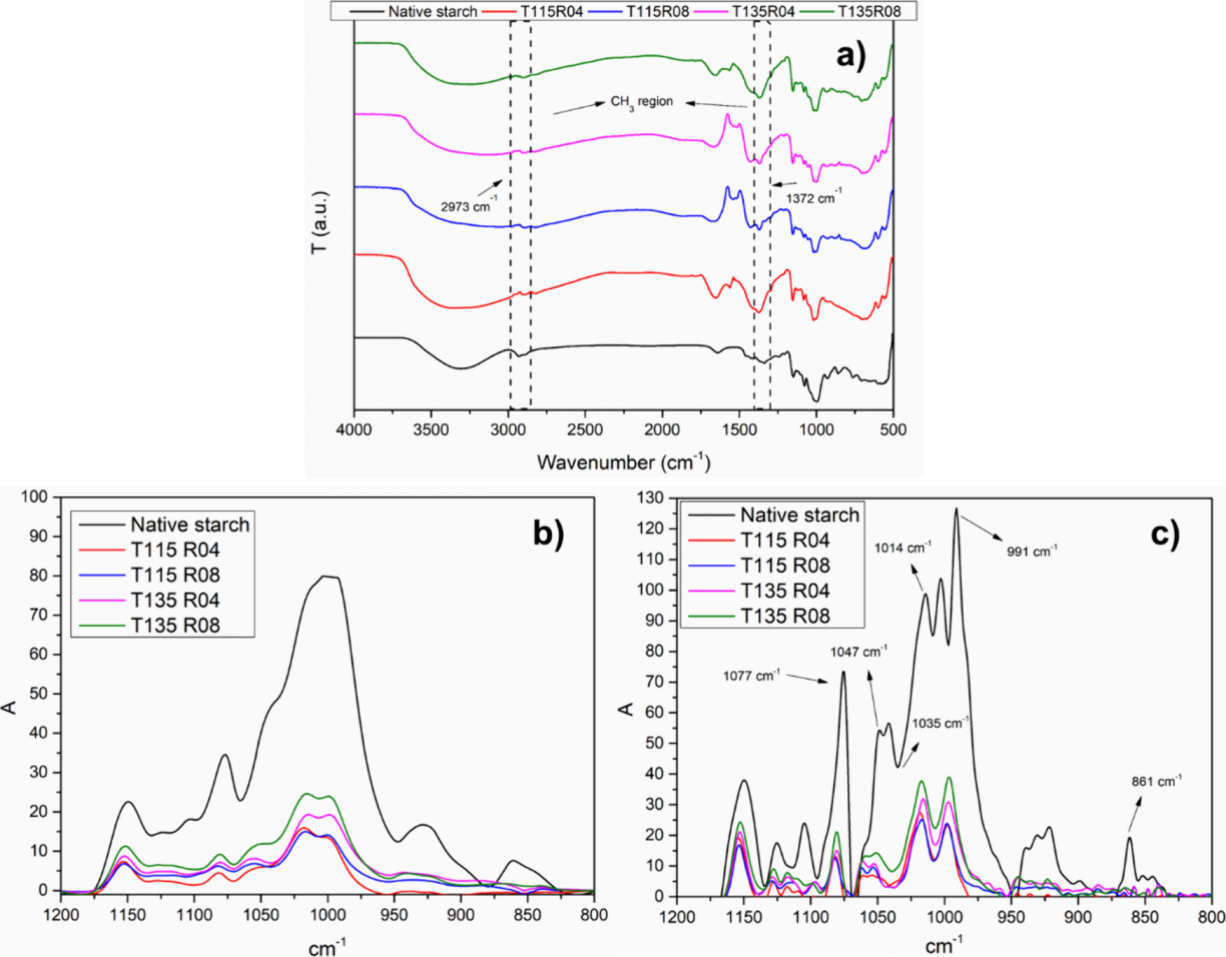
FTIR complete spectra (a) of all samples (see Table 1 for designations). Original (b) and deconvoluted spectra (c) of the fingerprint region of starch.

After modification with PO in the autoclave reactor, all samples exhibit an absorption band at 2973 and mainly at 1372 cm^–1^, related to methyl absorption, as observed in Figure 1a, confirming the extent of modification. This peak is not observed for native starch.

Compared to FTIR spectra of native starch, all modified samples showed only slight differences in absorption bands around the 3400–3000 cm^–1^ region, assigned to the O–H stretching vibration (Figure 1a). Literature findings mention alterations in HPS hydrophobicity and may explain this observation,^5^ alongside the grafting of PO groups and KOH activation, which occurs in the hydroxyl region. The distinctive feature observed is the aliphatic C–H stretching regions, marked at 1372 cm^–1^, but also present at 2973 cm^–1^, with the appearance of an absorption band linked to methyl groups (CH_3_) grafted after PO modification.^37,38^ The strong peak at 1372 cm^–1^ is also linked to methyl groups and is observed by others, emphasizing the extent of the modification.^5,13,14,39^ Ether moieties located at 1100–1000 cm^–1^ also showed some alterations, as expected, by the modification of hydroxyl groups by PO molecules and seems to be more marked at higher temperatures and/or molar ratios.^5,39^

Short-range quantitative analysis was performed in processed FTIR spectra, presented in Figure 1b,c. After modification, all bands located in the fingerprint region of starch between 1200 and 800 cm^–1^ presented a significant decrease in intensity, indicative of B-type lattice loss of order at a molecular level,^40^ promoted by hydroxypropylation. According to the authors,^34^ bands located in 1047, 1022, and 861 cm^–1^ are sensitive to changes in the crystallinity, clearly seen in Figure 1 b. Well-defined bands located at the 1022 cm^–1^ region are obtained for all modified materials, which may indicate a higher amount of ordered short-range double-helix structures.^40^ Increased ordering at a molecular level reflects in a more intense band located at 1047 cm^–1^, allied to a shift in band typically observed at 994 cm^–1^, caused by different amounts of water.^34^ The same shift occurs to the band located at 1022 cm^–1^, as observed in Figure 1c.

**Figure 2:**
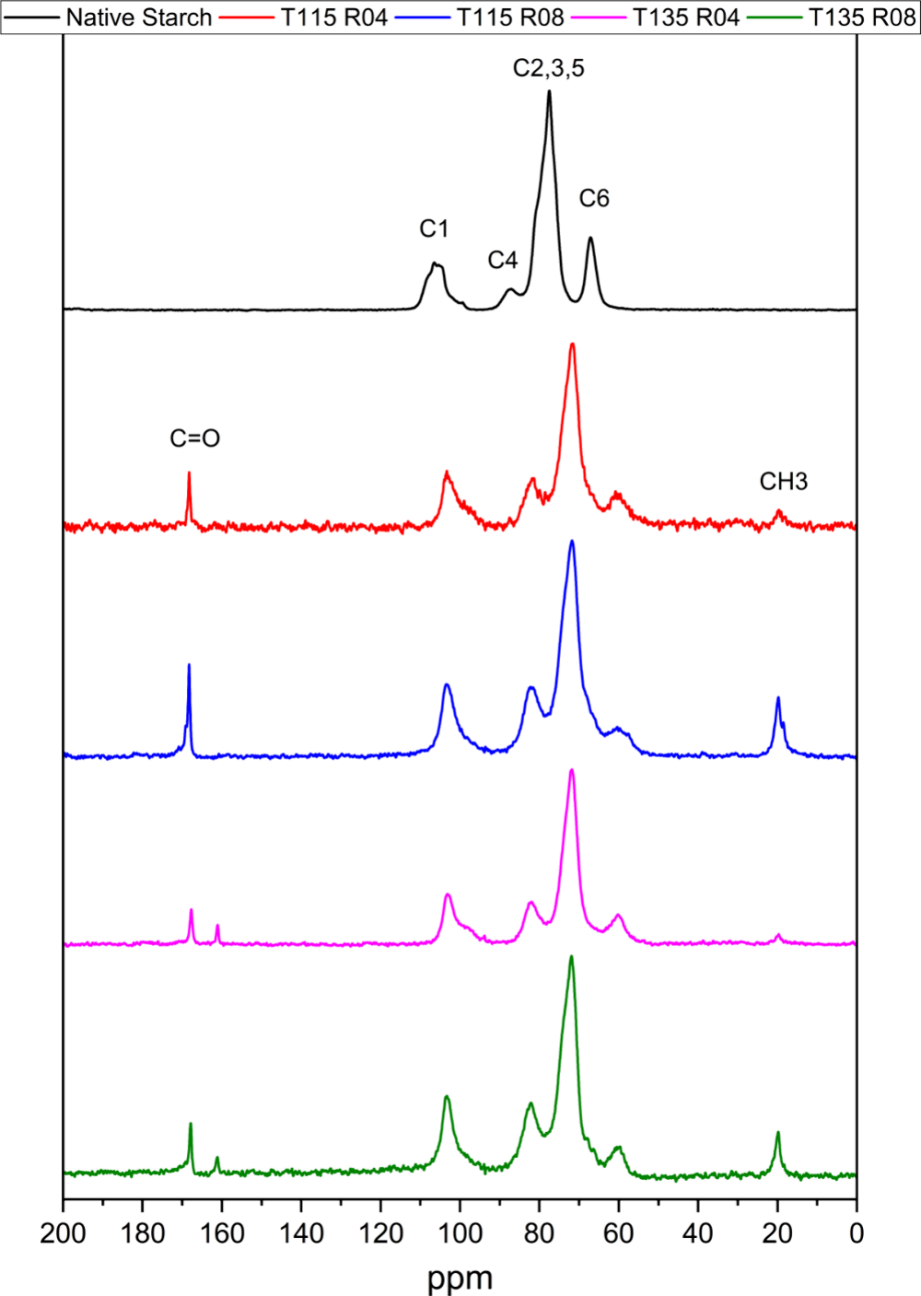
^13^C NMR spectra with signal assignments for native starch and modified samples.

^13^C NMR analysis provides valuable insights into the process of modifying native starch with PO. Figure 2 clearly shows the successful introduction of the PO group into the starch structure. This is confirmed by the presence of a new signal around 20 ppm, which is characteristic of the methyl group (CH_3_) introduced by the PO, likely through ether linkages, also observed by others.^35,41−43^ Furthermore, considering chemical shifts, it can be inferred that the modification occurred most likely at C2 and C3.

In addition, higher molar ratios of PO (0.8) led to significantly increased DS compared with lower molar ratios (Table 3). This indicates that a greater availability of PO molecules enhances the modification process.^38^ Interestingly, lower reaction temperatures (115 °C) resulted in higher DS for both molar ratios compared to the higher temperature (135 °C), as observed also in mass gain results. This suggests that excessive heat might be detrimental to the modification process, potentially leading to side reactions or the degradation of the starch structure. In addition, the DS values reported in this work are similar to those observed by ref (35), considering that the reaction procedures used by the authors are very similar to this work, with a greater amount of PO. Also, it must be considered that in the latter work, the authors modified a cellulose structure, which is less available to modification compared to starch due to its less free volume near hydroxyl groups.

**Table 3:** CNH and O Relative Content of O Obtained by Elemental Analysis

sample	%C	%H	%O	H/C	DS_EA_	DS_NMR_
native starch	39.58	6.67	48.21	0.168		
T115 R04	26.10	5.12	47.60	0.196	0.16	0.15
T115 R08	24.49	5.32	47.70	0.217	0.18	0.45
T135 R04	27.78	5.04	45.48	0.181	0.15	0.10
T135 R08	26.20	4.74	43.67	0.181	0.16	0.28

MS values observed for sago starch presented an increase with increasing PO volume,^38^ similar to the trend observed in this work. An additional finding was the presence of a carbonyl group in two different signals at the region of 168 ppm in the modified starch samples, commonly observed only in esterification reactions,^35,43^ being attributed to ester linkages and carboxylic acid.^43^ While the exact origin of this signal requires further investigation, it might be attributed to some degree of starch degradation during the modification process. This potential degradation could be a consequence of the reaction conditions, particularly higher temperatures.

In conclusion, the analysis effectively demonstrated the successful chemical modification of starch with PO. The DS can be controlled by adjusting the molar ratio of PO, and the reaction temperature seems to have a complex influence, potentially affecting both the substitution and degradation processes.

### 3.3 EA

The results of EA are displayed in Table 3 for all modified samples and for native starch. The carbon content of all samples was lower than that of the control sample, especially for samples obtained with a higher molar ratio (0.8). The hydrogen content presented a singular behavior, different from the one observed for carbon, where higher temperatures resulted in lower hydrogen content. The results obtained are different from those observed for HPS,^44^ chitosan,^45^ and cellulose^36^ in the literature, where hydroxypropylation or other modification increased both carbon and hydrogen contents.

No nitrogen or sulfur was identified in any samples, and the sum of each element may not result in 100% due to the presence of potassium, which was not quantified. Proportionally to carbon, hydrogen content increased for modified samples, as indicated by the H/C ratio, which may indicate the chemical modification, due to the insertion of a methyl group (CH_3_) that increases the hydrogen content per carbon in the anhydroglucose unit. Corroborating with FTIR and mass gain outputs, DS values present the substitution extent of HPS in addition to the observation that lower temperatures allied to higher molar ratios facilitate the modification. Despite the variations in DS values obtained from each technique, both methods exhibit the same overall trend, where lower temperatures and higher molar ratios produced higher DS values, as seen in ref (46). Compared to surface modification of cellulose, DS values presented in Table 3 are half of those obtained by other authors^36^ and similar to those obtained by ref (45), with a lower amount of epoxide used to modify chitosan materials. Etherified pullulan materials presented significantly higher values of DS, around 1.0–2.5, with similar molar ratios, varying from 0.66 to 2.00.^47^

### 3.4 SEM

**Figure 3:**
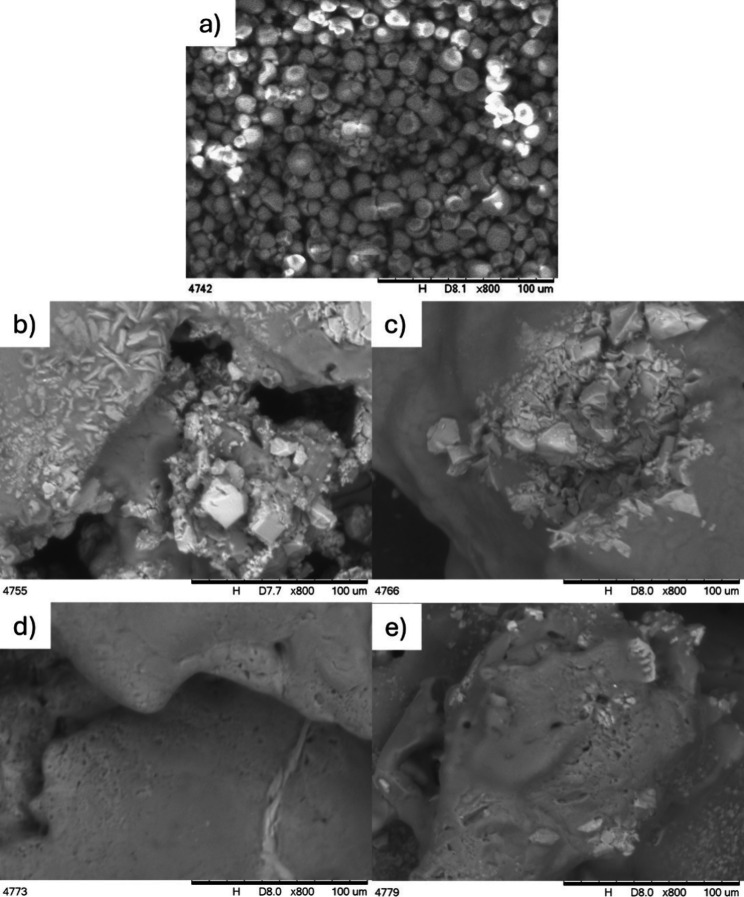
SEM images of native starch (a) and modified samples T115 R04 (b), T115 R08 (c), T135 R04 (d), and T135 R08 (e).

Figure 3 shows the SEM morphology of native starch and modified samples (varied magnifications are shown in the SI). A few solid particles are observed, mainly for samples obtained at lower temperatures (115 °C), and are linked to the presence of the potassium residue.^48,49^ SEM analysis shows that unlike native starch, no granular structure is observed for modified samples. This may be related to the complete disruption of starch granules during the reaction in the presence of PO and under high temperatures. This observation contrasts with what has been reported by others in the literature^5,17,22,37,38,50^ but is partially similar to those observed by other authors,^16,26,27,51^ considering that in some cases, the gelatinization occurred only partially and/or under high shear and temperature conditions. Also, it is worth noticing that in most of these works, starch’s hydroxypropylation was not performed under high temperature and pressure, which can result in difficult granule disruption. Kaur and coauthors mentioned that the hydroxypropylation reaction may facilitate granular disruption by introducing PO groups and weaken the intragranular interactions, reflecting an increased motion of amorphous portions within the starch structure.^19^ The plasticization effect induced by PO was already observed for starch and is discussed elsewhere.^16,26,27^

### 3.5 X-ray Diffraction

**Figure 4:**
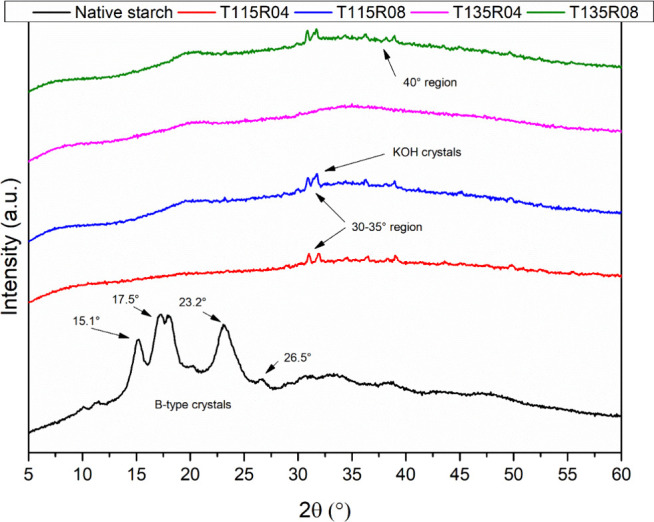
XRD patterns are for native starch and modified samples.

The disruption of starch granules upon chemical modification was also evidenced by XRD analysis, as shown in Figure 4. No crystalline structure was detected for modified samples, while native starch presented the typical B-type structure of tuber starch, as comprehensively described by refs (8,10,11), presenting a crystallinity index of 45.2%.

Typical B-type structure peaks were identified at around 15.1, 17.5, 23.2, and 26.5° only for native starch.^8,10^ No crystalline peaks were identified for modified samples, confirming the gelatinization of starch during the reaction, contrary to what was observed by ref (52) where the authors state that the main portion modified by hydroxypropylation was the amorphous domain. Crystals identified at 30–35 and near 40° are related to the presence of residual potassium from the activation process, which was insoluble in hexane during Soxhlet extraction. Potassium compounds are commonly detected in these regions.^48,49,53,54^ The amount of short-range ordering in starch is expressed by the height ratio at 1047 and 1035 cm^–1^, called *R*(1047/1035), in Table 4.

**Table 4:** Short- and Long-Range Order Values Expressed by *X*_C_ and *R*_(1047/1035)_ and *R*_(1047/1014)_

sample	*X*_C_ (%)	*R*_(1047/1035)_	*R*_(1047/1014)_
native starch	45.2	1.28	0.55
T115 R04		1.87	0.25
T115 R08		3.31	0.38
T135 R04		3.67	0.33
T135 R08		1.72	0.38

Long-range orders are absent in all modified materials investigated by XRD, due to the inobservance of any crystalline peaks. However, few short-range orders are present in the form of double-helical amylopectin structures, due to lower values of *R*_(1047/1035)_ and higher *R*_(1047/1014)_ values, in agreement with the discussed in literature.^40,55,56^

### 3.6 DSC and TGA

**Figure 5:**
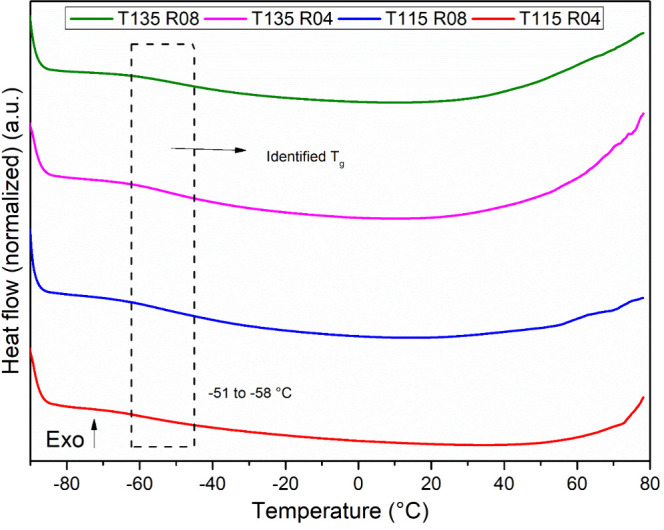
DSC curves for the modified samples.

The thermal properties of all modified samples were investigated by DSC, and the results are presented in Figure 5. Native starch showed a gelatinization temperature of 68.3 °C with an enthalpy of 3.13 J g^–1^ (shown in the SI).

DSC curves corroborate the observations made by SEM analysis, showing no endothermic peak of gelatinization, indicating that it had already occurred during the reaction. This contrasts with several literature findings, where authors did not obtain gelatinized starch after hydroxypropylation.^17,23,27,38^ Additionally, slight changes in the baseline were identified around −51 to −58 °C for all modified samples and are related to the glass transition temperature (*T*_g_) of residual PPO, as comprehensively described by refs (14,18) even after extraction, which is a clear indication of PPO formation during the reaction. It is important to note that, after 12 h of Soxhlet extraction with *n*-hexane, the amount of polyol recovered from the bottom of the system was not sufficient for its characterization, indicating that it may be well grafted onto the starch backbone.

**Figure 6:**
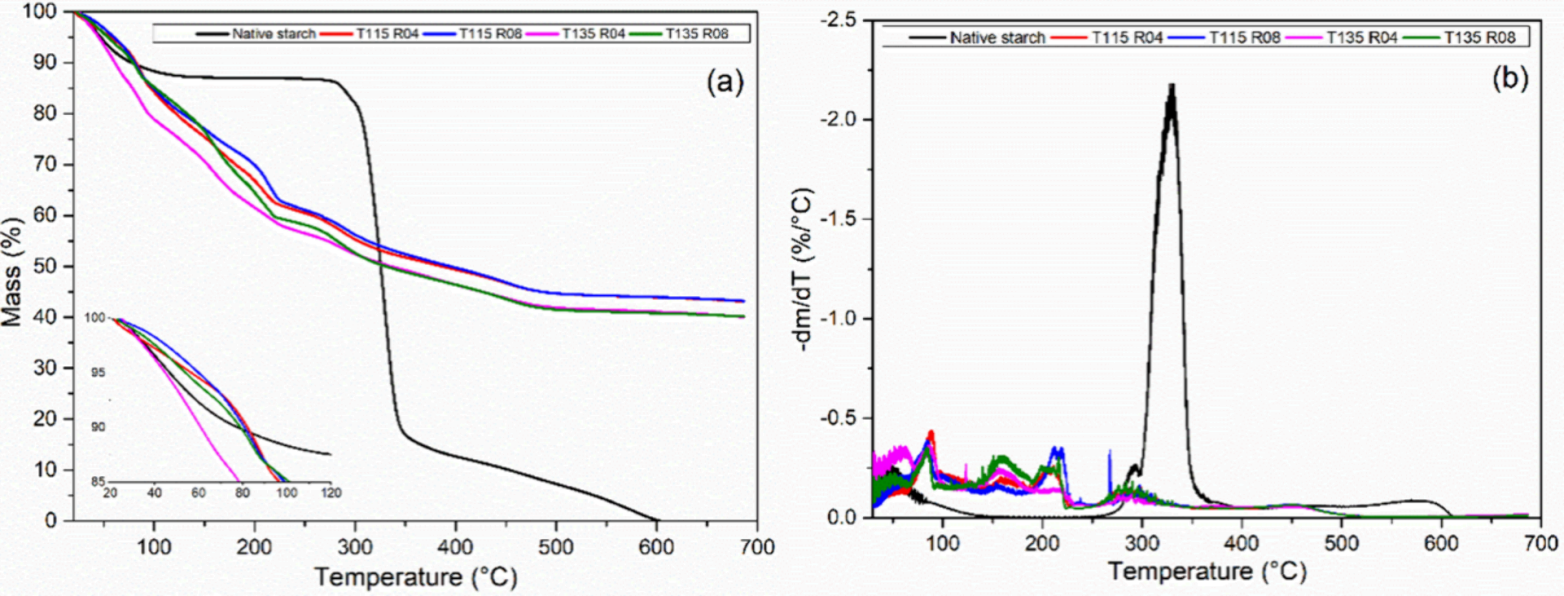
TG (a) and DTG (b) curves for native starch and modified samples. The inset in (a) is an expanded view of the dehydration stage.

Results from TGA are listed in Figure 6.

All modified samples demanded higher temperatures to dehydrate when compared to native starch, except for T135 and R04. Alterations in dehydration behavior are observed in Figure 6a and were also observed by others, indicating that hydroxypropylation played a role in this feature.^5^ Heating resulted in several degradation steps for modified samples, regardless of temperature and molar ratio, clearly seen in the DTG curve in Figure 6b. This indicates the presence of residual PPO, previously identified by DSC, reducing the material’s decomposition temperatures, due to its low molecular weight and poor thermal stability.^18^ This behavior was also observed for hydroxypropylated cellulose.^12,13^ A residual inorganic mass of around 40% was observed in TGA experiments, which is linked to the presence of potassium, already observed in SEM images and XRD.

Differences in *D*_5%_ and *D*_15%_ showed no trend related to the parameters used, as described at Table 5, with up to 8.9% of increase between modified samples and 11.7% compared to native starch, mainly caused by the presence of residual inorganic compounds. At *D*_50%_, significant increases of 18.9 and 21.4% in decomposition temperature are observed for the T115 pair, also related to the presence of potassium, also discussed in terms of residue percentage and observed in SEM analysis.

**Table 5:** Decomposition Temperatures of Each Sample Analyzed by TG^a^

sample	*D*_5%_ (°C)	*D*_15%_ (°C)	*D*_50%_ (°C)	*R*_700°C_ (%)
native	46.88	95.60	325.35	0.00
T115R04	48.09	96.72	386.78	43.11
T115R08	50.66	99.38	394.89	43.21
T135R04	52.38	101.01	335.91	40.07
T135R08	49.29	98.04	330.56	40.15

a D = decomposition. R = residue.

According to the results obtained, we believe that hydroxypropylation of starch occurred not only in the amorphous phase but also in the crystalline phase, by the homopolymerization of PPO molecules concomitantly to the grafting of PO during the reaction, a feature that was also discussed by others.^23^ This caused the disruption of the granule and, consequently, the gelatinization of starch, supported by high temperature, pressure, and pH used during the process. It is known that hydroxypropylation of starch takes place in the amorphous and central regions of the granule, due to its higher free volume and less steric hindrance,^24^ but the results obtained in this study lead to the conclusion of occurrence also in the crystalline region by its inobservance in several analysis. Considering PPO homopolymerization, starch structure may be disrupted during its chain growth and transfer, under high temperature (above gelatinization) and pressure, in the presence of low molecular weight compounds. PPO molecules were detected by TGA, under several steps of mass loss, and by DSC, with the detection of a *T*_g_ near −58 °C.

## 4 Conclusions

Gelatinized cassava starch was obtained directly by the hydroxypropylation reaction performed in an autoclave reactor under high pressure and defined temperatures of 115 and 135 °C, mostly different from the observations reported in the literature related to PO reactions. This gelatinization was obtained by the homopolymerization of PPO within starch granules during the reaction. Using ANOVA, the parameters defined resulted in different mass gains of the modified materials, where lower temperatures and higher molar ratios promoted higher mass gains. Gelatinization was confirmed by the absence of endothermic peaks in the DSC curves and by the absence of granular structures in the SEM images. The observation of a predominantly amorphous phase by XRD for all modified samples confirms these findings, despite the presence of short-range order structure identification via FTIR. Additionally, FTIR, NMR, and EA confirmed the extent of modification by the appearance of methyl group signals and high DS values, allied to the presence of low molecular weight molecules (PPO), detected in TGA and DSC. The remaining challenges of this work are related to the complete removal of reaction components, such as potassium, unreacted PO, and/or excess of PPO, which was not achieved through Soxhlet parameters used. Also, a lower KOH molar ratio and a method to control PPO homopolymerization and/or grafting reactions, which occur in parallel, are desirable and directly related to the parameters used in this work. Nonetheless, it is clear that the presence of PPO plays a role in starch thermal stability, decreasing it by the plasticization effect caused, increasing motion of starch chain segments. The presence of a plasticizing molecule within starch chains after the reaction can be an environmental benefit, by decreasing the quantity or even dispensing the necessity of synthetic plasticizer addition in further processing, such as casting or extrusion.

## Supporting Information Available

Additional experiments of amylose content and molecular weight of starch, reaction pressure per time curve, native starch gelatinization results, and statistical analysis (PDF)
