# Supplementary material for: Gelatinized Cassava Starch Obtained via Low Molar Ratio Hydroxypropylation Reaction
Source: ACS Omega. 2025 Mar 20;10(12):12543–52. doi: 10.1021/acsomega.5c00246 (PMC11966575; doi:10.1021/acsomega.5c00246)
Supplement: Supplementary file 1 — ao5c00246_si_001.pdf [file ao5c00246_si_001.pdf]

GELATINIZED CASSAVA STARCH OBTAINED VIA LOW MOLAR RATIO HYDROXYPROPYLATION REACTION

Henrique Solowej Medeiros Lopes[1,2,5]*, Fernanda Andrade Tigre da Costa[3,5], Daniel Komatsu[2,4], Alain Dufresne[5] and Aparecido Junior de Menezes[1]

[1]Federal University of São Carlos (UFSCar), 110 km João Leme dos Santos Road, Sorocaba, SP, 18052-780, Brazil.

[2]Technological College of Sorocaba (Fatec), 2015 Carlos Reinaldo Mendes Avenue, Sorocaba, SP, 18013-280, Brazil.

[3]Nuclear and Energy Research Institute (IPEN-CNEN/SP), 2242 Prof. Lineu Prestes Avenue, São Paulo, SP, 05508-000, Brazil.

[4]Pontifical Catholic University (PUC), 290 Joubert Wey St., Sorocaba, SP, 18030-070, Brazil.

[5]Univ. Grenoble Alpes, CNRS, Grenoble INP, LGP2, F-38000 Grenoble, France

*E-mail: henrique.lopes01@fatec.sp.gov.br

Supporting information

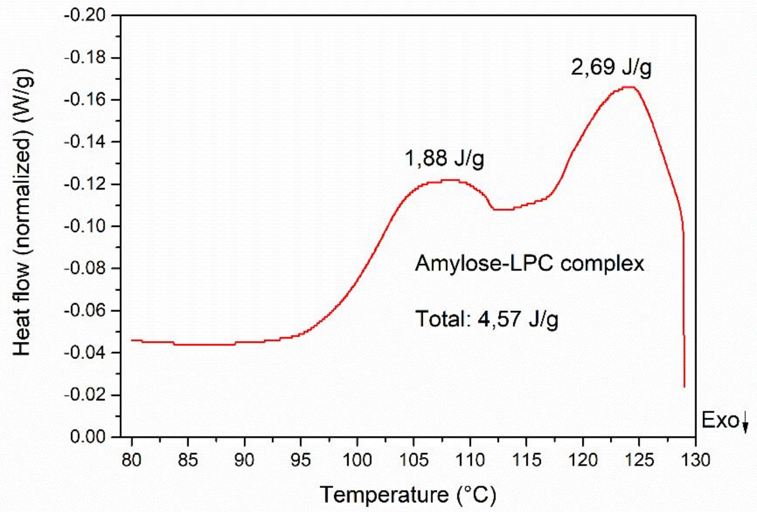

Figure S1. amylose-LPC complex, measured for the food grade cassava starch of this work, according to the described in [1–3].

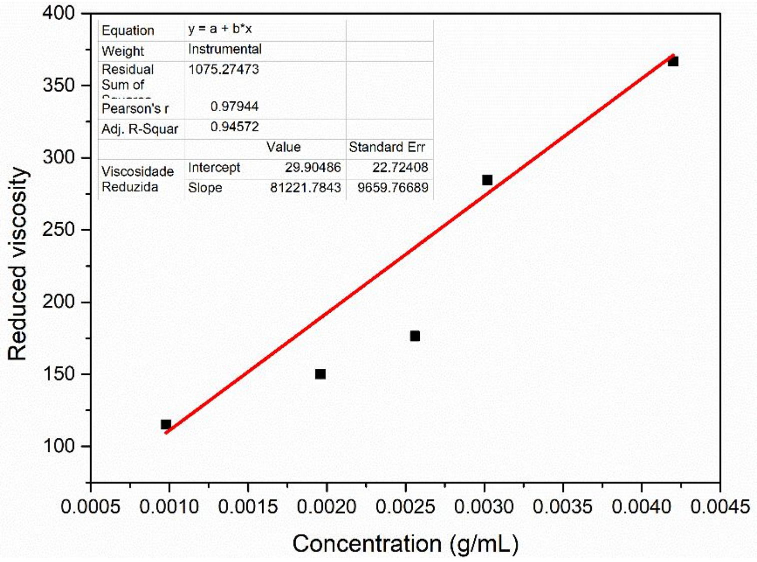

Figure S2. reduced viscosity versus concentration plot used for linear regression on molecular weight calculation of starch, according to a previous methodology described in [4].

Table S1. flow times measured by intrinsic viscosity measurements

Concentration (g/mL)	t1	t2	t3	Average	Standard deviation
Solvent	151.98	151.99	151.63	151.87	±0.20
0.00052	165.20	164.13	164.53	164.62	±0.54
0.00098	169.57	168.06	169.41	169.01	±0.83
0.00196	195.93	195.27	198.39	196.53	±1.64
0.00256	218.34	218.78	224.28	220.47	±3.31
0.00302	282.78	281.34	282.87	282.33	±0.85
0.00420	385.30	388.30	383.81	385.80	±2.29

0.00524	397.65	395.68	392.11	395.15	±2.81

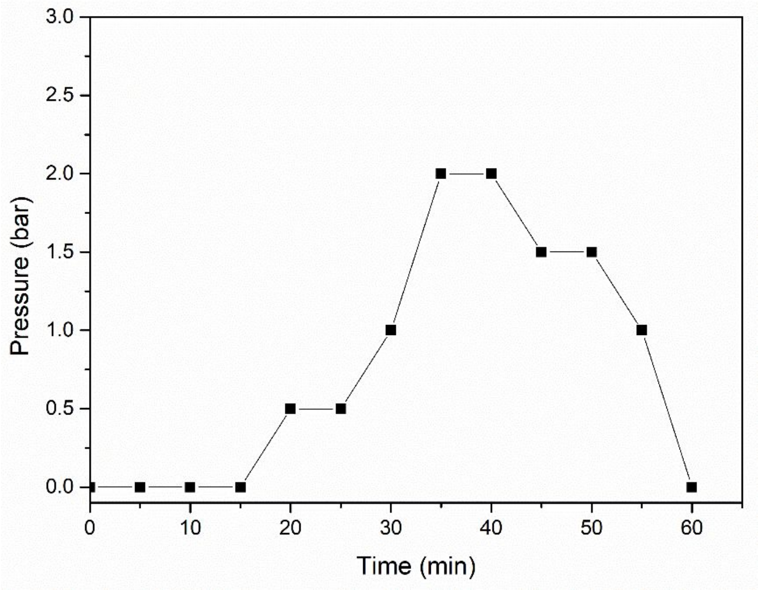

Figure S3. Typical pressure per time curve of the hydroxypropylation reaction performed. All materials presented similar behavior during the reaction, with slight variations.

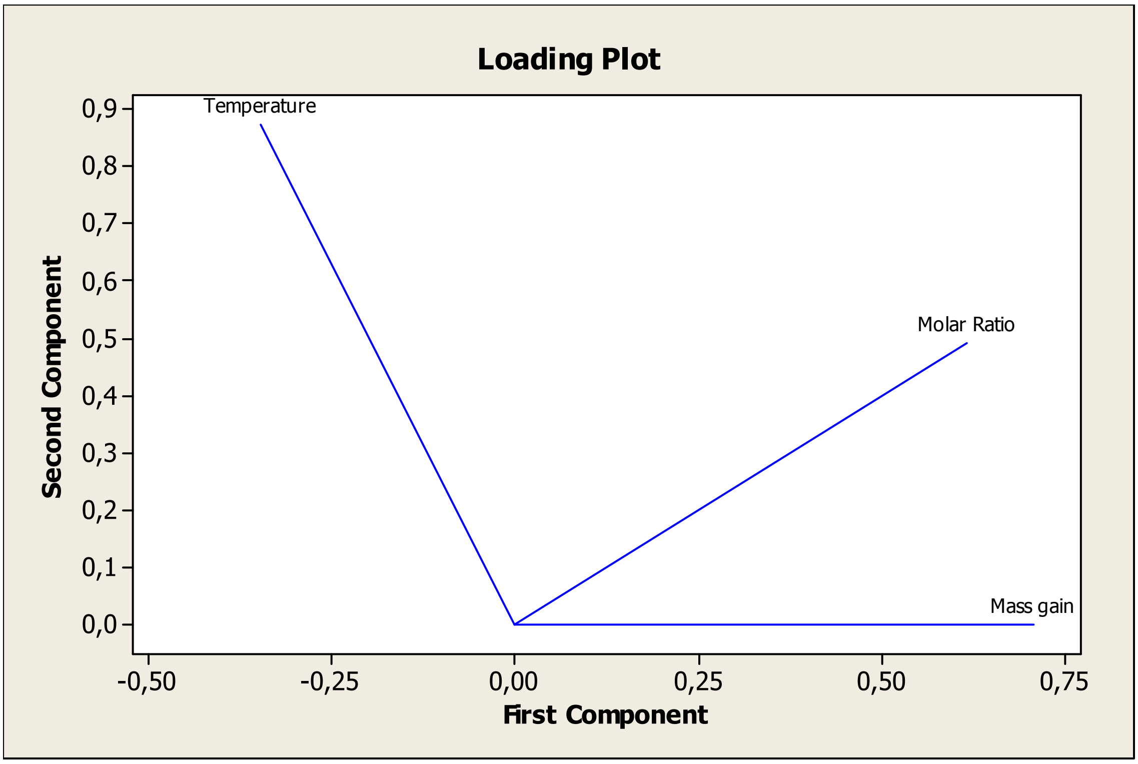

Figure S4. Loading plot of first and second component.

Table S2. Principal component analysis of the dependent and independent variables

Variables	PC1	PC2	PC3
Temperature	-0.347	0.871	0.347
Molar ratio	0.616	0.491	-0.616
Mass gain	0.707	0.000	0.707

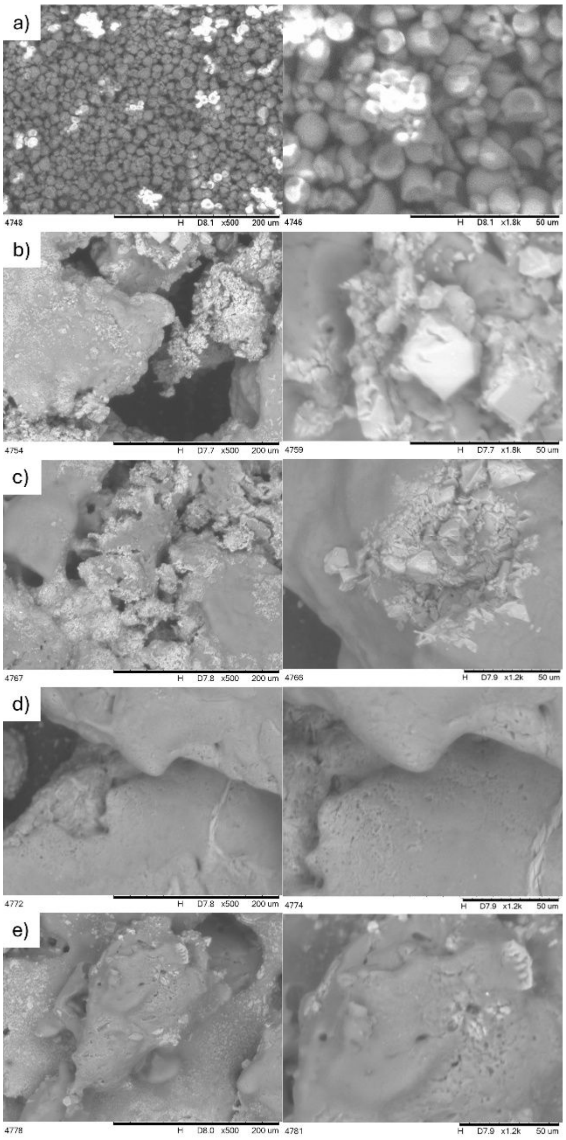

Figure S5. SEM images with varied magnification of native starch (a) and modified samples T115 R04 (b), T115 R08 (c), T135 R04 (d), and T135 R08 (e).

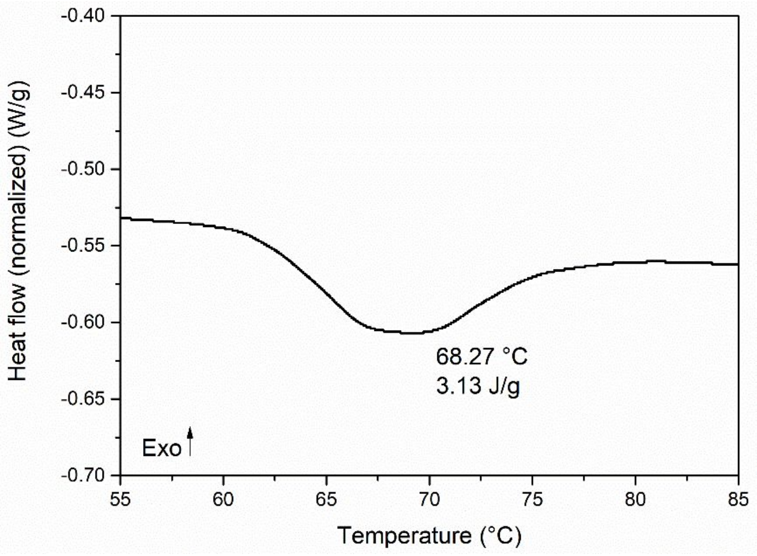

Figure S6. Native starch gelatinization temperature and enthalpy.

References

1. Mestres C, Matencio F, Pons B, et al. A Rapid Method for the Determination of Amylose Content by Using Differential-Scanning Calorimetry. Starch/Stärke 1996;48(1):2–6.
2. Kugimiya M, Donovan JW. Calorimetric Determination of the Arnylose Content of Starches Based on Formation and Melting of the Amylose-Lysolecithin Complex I. J Food Sci 1981;46:765–776.
3. De Morais Teixeira E, Da Róz AL, De Carvalho AJF, et al. Preparation and Characterisation of Thermoplastic Starches from Cassava Starch, Cassava Root and Cassava Bagasse. In: Macromolecular Symposia 2005; pp. 266–275; doi: 10.1002/masy.200551133.
4. Lopes HSM, Oliveira GHM, Talabi SI, et al. Production of thermoplastic starch and poly (butylene adipate-co-terephthalate) films assisted by solid-state shear pulverization. Carbohydr Polym 2021;258; doi: 10.1016/j.carbpol.2021.117732.
